# A systematic review of factors impacting intraoral scanning accuracy in implant dentistry with emphasis on scan bodies

**DOI:** 10.1186/s40729-024-00543-0

**Published:** 2024-05-01

**Authors:** Peter Gehrke, Mahsa Rashidpour, Robert Sader, Paul Weigl

**Affiliations:** 1 Department of Postgraduate Education, Master of Oral Implantology, Center for Dentistry and Oral Medicine (Carolinum), Johann Wolfgang Goethe University, Theodor-Stern-Kai 7, 60596 Frankfurt, Germany; 2 Private Practice for Oral Surgery and Implant Dentistry, Bismarckstraße 27, 67059 Ludwigshafen, Germany; 3 Master of Oral Implantology, Center for Dentistry and Oral Medicine, Johann Wolfgang Goethe University, Frankfurt, Germany; 4 Private Practice for Implant dentistry and Prosthodontics, Tehran, Iran; 5 Department for Oral, Cranio-Maxillofacial and Facial Plastic Surgery, Medical Center of the Goethe University, Frankfurt, Germany; 6 Head of Department of Postgraduate Education, Master of Oral Implantology, Center for Dentistry and Oral Medicine (Carolinum), Johann Wolfgang Goethe University, Theodor-Stern-Kai 7, 60596 Frankfurt, Germany

**Keywords:** Dental, Implant, Scan body, Accuracy, Intraoral scanning

## Abstract

**Purpose:**

The purpose of this systematic review was to explore and identify the factors that influence the accuracy of intraoral scanning in implant dentistry, with a specific focus on scan bodies (ISBs).

**Methods:**

Following the PRISMA 2020 guidelines, this study conducted a thorough electronic search across MedLine, PubMed, and Scopus to identify relevant studies. Articles were screened based on titles, abstracts, and full texts for relevance. The Robins I tool assessed the risk of bias in various study types. Data extraction occurred based on predetermined parameters for studying specimens and assessing outcomes.

**Results:**

16 studies met the specified criteria and were consequently included in the systematic review. Due to variations in variables and methods across the selected studies, statistical comparison of results was not feasible. Therefore, a descriptive review approach was chosen, acknowledging the substantial heterogeneity in the reviewed literature.

**Conclusions:**

The precision of virtual scan results is contingent upon diverse characteristics of ISBs and implants. These factors encompass their placement within the dental arch, structural design, shape, material composition, color, and the manufacturing system, all of which contribute to scan accuracy. Additionally, considerations such as the intraoral scanner (IOS) type, scanning technique, use of scan aids, inter-implant distance, scan span, and the number of implants warrant evaluation. In the context of capturing implant positions, intraoral scanning with ISBs demonstrates comparable accuracy to traditional impression methods, particularly in single and short-span scenarios. However, the existing data lacks sufficient information on in vivo applications to formulate clinical recommendations.

**Supplementary Information:**

The online version contains supplementary material available at 10.1186/s40729-024-00543-0.

## Graphical abstract

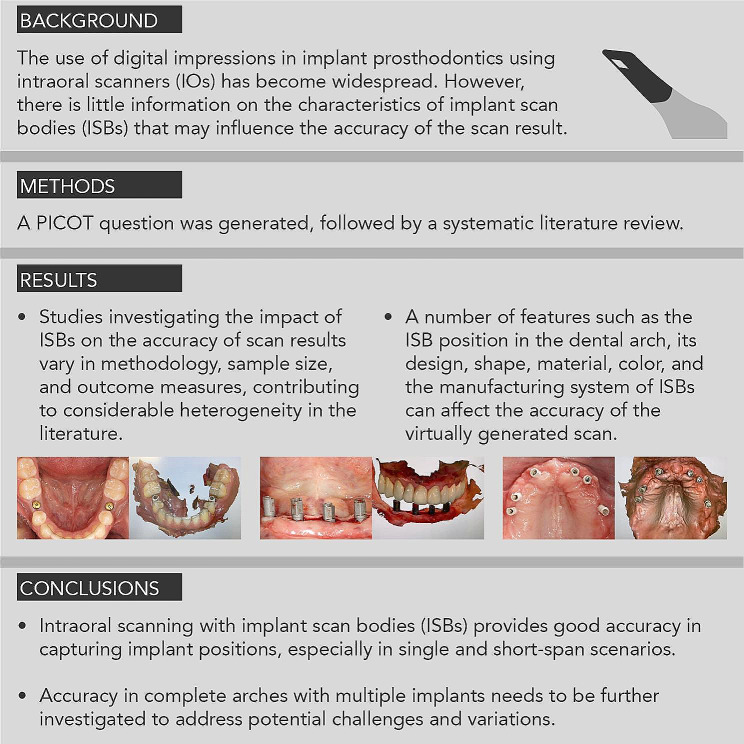

## Introduction

Over time, implant-supported restorations, clinically validated for successfully restoring missing teeth [1], have shown to enhance masticatory function, positively influence nutritional well-being, and contribute to an elevated overall quality of life and patient satisfaction compared to conventional dentures [2, 3].

**Fig. 1:**
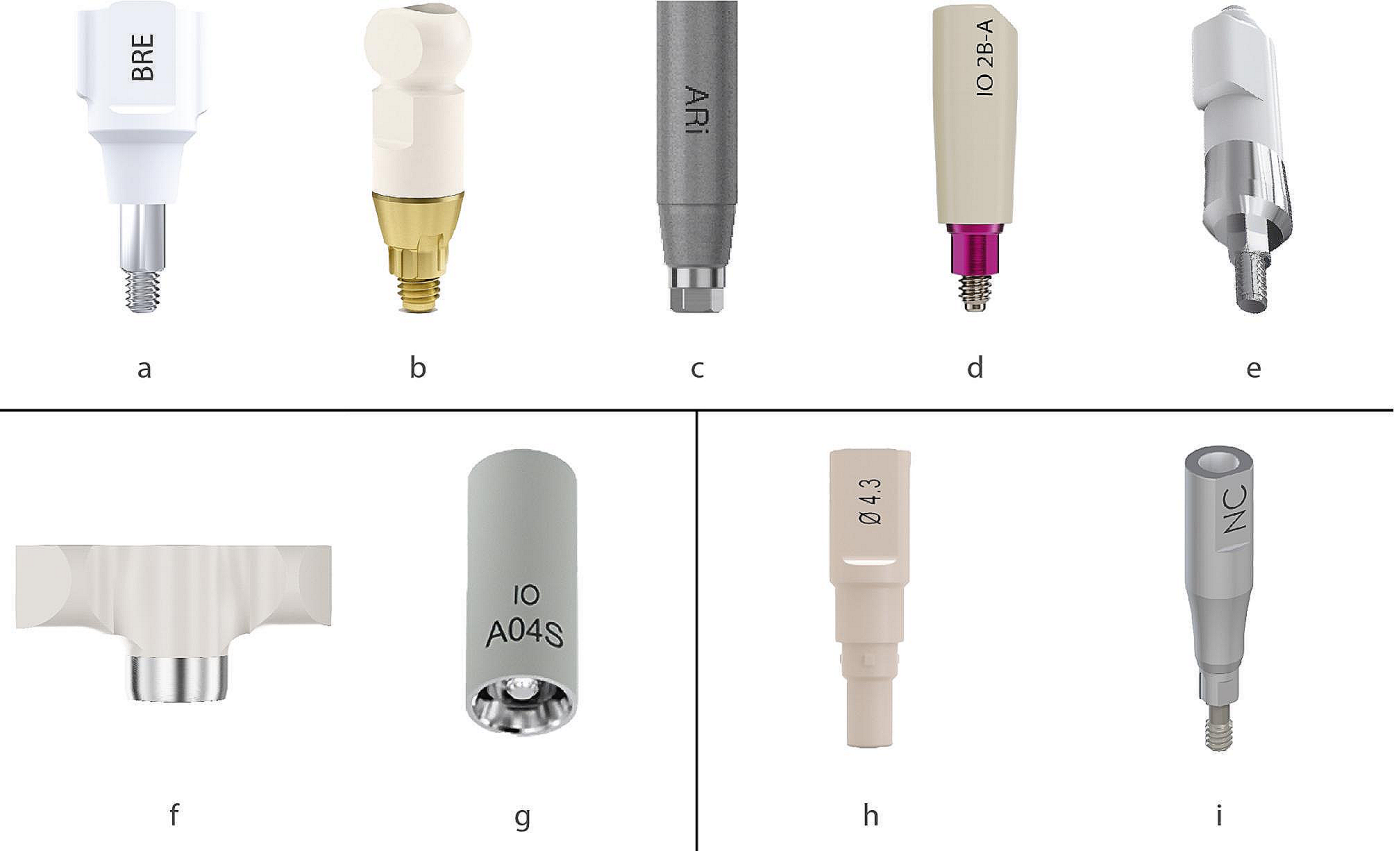
Examples of different design and material options for industrially available implant scan bodies (ISBs) from left to right. Group a-e: Two-piece ISBs for scanning single or multiple adjacent implants, consisting of polyetheretherketone (PEEK) or resin on the upper scanning portion and a metal base to be inserted into the implant connection. (**a**) Sky scan body Bredent Medical, Senden, Germany; (**b**) Atlantis IO FLO, Dentsply Sirona Implants, Mölndal, Sweden; (**c**) AnyRidge Scan-Abutment, MegaGen, Daegu, Korea; (**d**) Elos Accurate Intra Oral Position Locator NP, NobelBiocare, Zurich, Switzerland; (**e**) Zimmer Intraoral scan body, ZimVie, Florida, USA. Group **f**-**g**: Two-piece ISBs (PPEK & metal base) for scanning and manufacturing of multi-unit implant supported superstructures. (**f**) Smart Flag, Apollo, Pabianice, Poland; (**g**) Atlantis IO FLO-S, Dentsply Sirona Implants, Mölndal, Sweden. One-piece ISBs in a single material such as PEEK: (**h**) Camlog scan body, Wimsheim, Germany or titaniumi) Straumann NC scan body Titan, Institut Straumann AG, Basel, Switzerland for scanning of single or multiple adjacent implants

Dental impressions are imprints of teeth, implants and the surrounding anatomical structures in the oral cavity used in restorative dentistry. These impressions can be obtained with both conventional and digital techniques. In the conventional impression method for implants, an impression coping is attached to the implant while the impression is made with an impression tray and a silicone base material. In the digital method, on the other hand, intraoral scan bodies (Scan bodies) are screwed to implant fixtures and intra-oral scanning (IOS) is used to generate virtual data of the implant position and surrounding structures [4]. The use of digital intra-oral scanners has become prevalent in recent years, empowering practitioners to provide accurate and high-quality patient care [5]. Digital impressions can create a 3D computer-generated model faster than conventional techniques without causing nausea and discomfort for patients [6]. Implant scan bodies (ISBs) are scannable implant impression copings requiring specific scanner devices and scanning technologies. In recent years, ISBs have become available from a variety of companies, offering different design and material (Fig. 1), and scanning systems.

The accuracy of ISBs is determined by trueness and precision, which specifies the deviation from the reference and the reliability of repeated assessment [7]. Accumulating evidence has evaluated different variables that can affect the scan accuracy, such as scan body design, scanning system, implant location, and operator skills [8–10]. However, there are limited numbers of reports discussing the features of implant scan bodies (ISBs) that can affect their accuracy in implant dentistry [11].

A systematic review of the evidence demonstrating their trueness and precision is still lacking. Therefore, the aim of this systematic review is to investigate and identify the factors impacting the accuracy of intraoral scanning, with a specific emphasis on scan bodies.

## Materials and methods

The systematic review was reported in accordance with the PRISMA (Preferred Reporting Items for Systematic Reviews and Meta-analyses) statement. A protocol was developed and registered in the International Prospective Register of Systematic Reviews (PROSPERO ID: 451,137).

### Objectives

This review aims to address the following focus question: “Which parameters impact the accuracy of digital models’ scan outcomes, with a specific emphasis on characteristics associated with scan bodies?”

### PICOT question

The below intervention, comparison, outcome, and time frame (PICOT) was established to address the specified focus question:

Intervention (I): Parameters affecting the accuracy of scanned digital models (e.g., scan body design, scan body materials, implant type, implant angulation).

Comparison (C): No direct comparator. Compared indirectly with the performance data of conventional implant impression technique.

Outcome (O): The primary outcomes include the accuracy of scanned digital models in terms of scan result (e.g., trueness, precision, linear, and angular).

Time (T): In vitro and clinical studies up to March 2023.

### Sources of information and search strategy

An electronic search of three databases was conducted to identify eligible studies: MedLine, PubMed, and Scopus. The publication time was restricted after 2015. The language or publication type was limited to English. The literature search was conducted in March 2023, using a combination of controlled vocabulary and free keywords: dental, implant, scan body, and accuracy. Additional reports were identified through a manual search of the bibliographies of all included studies and relevant systematic reviews. The search strategy for each database was established as follows: (“dental implant” OR “dental implants”) AND (“scan body” OR “scan body”) AND (“accuracy” OR “precision” OR “reproducibility”).

### Eligibility criteria

#### Inclusion criteria

- The intervention should be relevant to the factors that may influence the accuracy of implant scan bodies in terms of the scan result.
- Randomized human clinical trials, controlled clinical trials, retrospective or prospective cohort studies, in vitro studies, and case series involving a minimum of 10 subjects (applies to clinical studies only).
- Studies published in English language.
- Articles reporting either on trueness or precision outcomes or both.
- If more than one article reported on the same study, only the article with the most recent results or the longest observation period was included in the analysis.

#### Exclusion criteria

- Studies only reporting animal findings.
- Case reports, abstracts only, protocols, book sections, conference proceedings, and narrative reviews.

### Study selection

Titles and abstracts identified in the search were screened independently by two reviewers (M.R., P.G.) using EndNote X9 software. If a title or abstract did not provide sufficient information on eligibility criteria, the full text was obtained. The full text was independently assessed by the same reviewers in order to select studies that met the eligibility criteria as described in Sect. 2.4. Open discussion between the two reviewers resolved any disagreements about eligibility during the process. Articles that did not meet the eligibility criteria were excluded. Reasons for exclusion were recorded.

### Data extraction

A digital data extraction sheet was developed in Excel software. One reviewer initially extracted the data from all the included articles, and the second reviewer double-checked all the proceedings. The author, year of publication, study design, ISB characteristics, IOS device, and factors influencing the accuracy were recorded for each included study. The parameters related to ISBs that affect the accuracy of intraoral scan outcomes were extracted accordingly.

### Quality assessment and risk of bias

The included studies were independently assessed by two reviewers for their methodological quality at the study level, and differences of opinion were resolved by discussion. A risk of bias quality assessment was performed using the ROBINS-I (Risk Of Bias In Non-randomized Studies - of Interventions) to assess the quality and potential bias of the included studies. The ROBINS-I tool was used for non-randomized studies [12, 13].

## Results

### Included studies

**Fig. 2:**
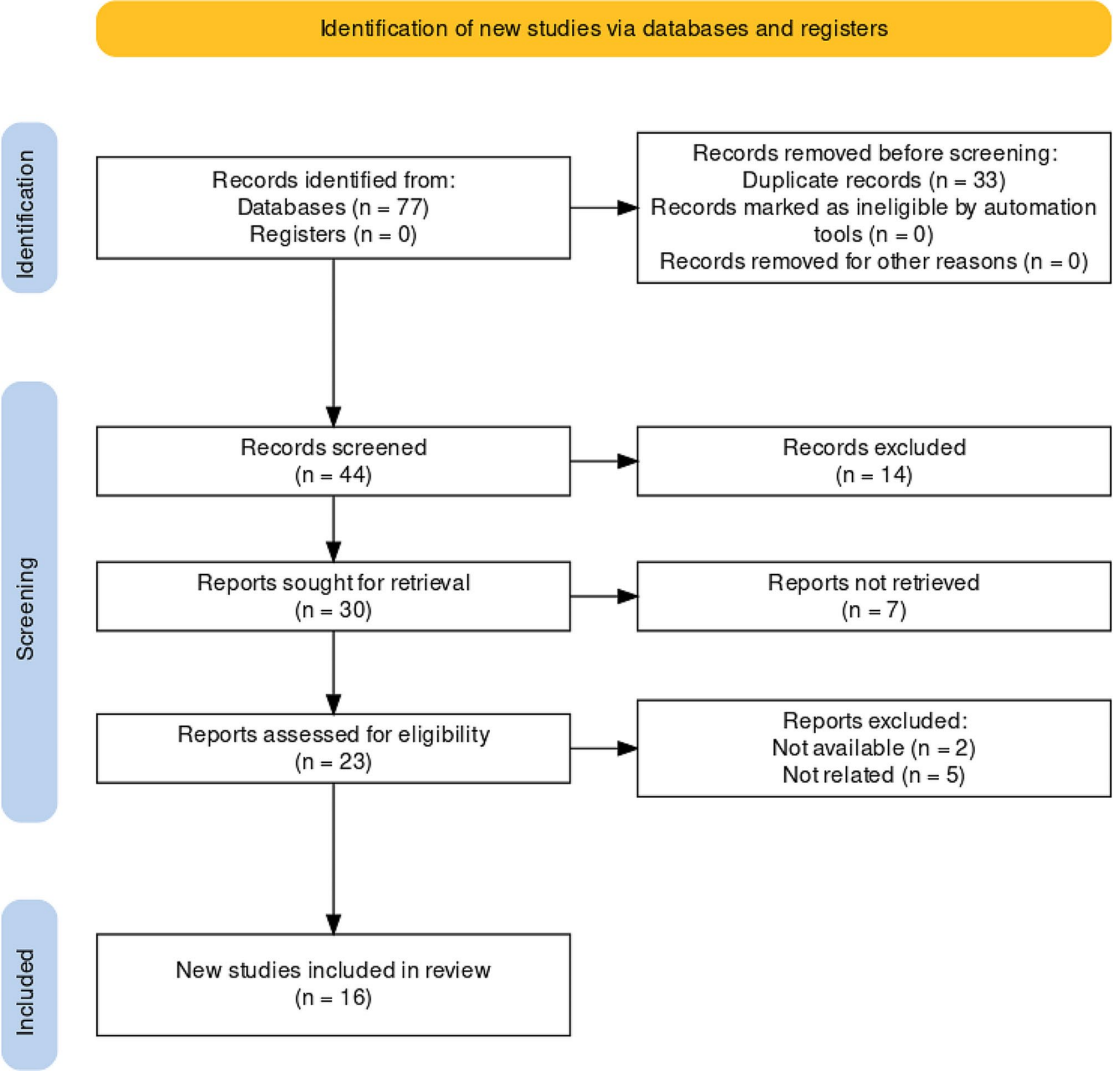
Flow chart of literature screening process

A total of 77 articles were initially identified from three different databases. After the removal of duplicates, 44 articles underwent a title and abstract screening. Subsequently, 23 articles were subjected to a full-text review, and 2 full-text articles were included for evaluation. Five articles were excluded for not meeting the eligibility criteria. Ultimately, 16 articles were deemed eligible. Figure 2 shows the flow chart of the screening process in the current study, generated using the PRISMA Flow Diagram tool [14]. A manual search was performed, but no additional articles meeting the inclusion criteria were found.

Of the 16 included studies, 13 were in-vitro investigations, and 3 were clinical investigations (level 2b) [15–17]. Notably, no randomized clinical trials (RCTs) were identified. The included studies explored various factors influencing the accuracy of digital implant scans. The factors investigated can be broadly categorized as follows: ISB Position- Influence of Palatal Area Stitching- Bevel Orientation, Placement, and Implant Angulation- Effect of Operator on Scan Precision- Effect of Scan Pattern- Impact of Implant Angulation and Depth- Effect of ISB Material- Comparison of Different IOS Devices- Scan Aids- Comparison of Digital and Conventional Impressions (Table 1).

**Table 1:** Summary of reviewed studies of factors affecting the accuracy of SBs

Author/Year	Type of study	Number of implants	Implant location	Scan body system	IOS device	Scanning system	Number of operators	Modification technique	Accuracy measurement criteria	Factors affecting accuracy
(33) Atalay et al., 2021	In-vitro	3	Left central incisor First premolar First molar	CHA-SB	TRIOS 3	ATOS Core 80	3	GOM Inspect	1-Distance and angular deviations 2-Operator performance	Implant location
[10] Giménez et al., 2015	In-vitro	6	27,25,22,12,15,17	-	CEREC	Manufacturer’s instructions	4	RE software	1-Distance and angular deviations 2-Operator performance	1-Inexprienced operator 1-Camera position
[10] Cakmak et al., 2022	In-vitro	1	Right first molar	SB, ScanPeg; Neoss Implant System, Harrogate, England	CEREC	ATOS Core 80 5MP	3	GOM Inspect	1-Circle-based technique 2-Point-based technique	Measurement technique
[20] Yilmaz et al., 2022	In-vitro	1	Right first molar	CHA-SB	TRIOS 3	ATOS Core 80	-	GOM Inspect	Distance and angular deviations	Scan patterns
[28] Bi et al., 2022	In-vitro	2–6	Right first molar Left first molar Left second molar	RN, WN; Straumann, Switzerland	TRIOS 3	D900	-	Geomagic Qualify 14 software	Distance and angular deviations	1-Implant location 2-Scanning distance
[18] Mizumoto et al., 2019	In-vitro	4	First molar First canine	MRM-DS	Trios 3	stereolithography	-	COMET L3D	Distance and angular deviation	Scan body position
[23] Mizumoto et al., 2020	In-vitro	4	First molar First canine	1-AF (IO-Flo; Dentsply Sirona) 2-NT (Nt-Trading GmbH & Co KG) 3-DE (DESS-USA) 4-C3D (Core3Dcentres) 5-ZI (Zimmer Biomet Dental)	Trios 3	1-unmodified master model 2-glass fiduciary markers placed on the edentulous ridge 3-pressure-indicating paste brushed over the ridge and palate 5-floss tied between the scan bodies	-	COMET L3D	1-Scan body system 1-Scanning technique	Scan body system and scanning technique
[24] Di Fiore et al., 2022	In-vitro	6	vertically and symmetrically at different heights into the master model	-	1-PrimeScan 2-Medit i500 3-Vatech EZ scan 4-iTero	Manufacturer’s instructions		Geomagic Studio Software	IOS devices	Primescan and iTero
[19] Gómez-Polo et al., 2022	In-vitro	4	Facial Mesial Distal Lingual Random	Avinent Transepithelial 4.8 scanbody	Trios 3	Manufacturer’s instructions	-	CAD software	1-Geometry bevel location 2-Implant angulation and position	1-Implant angulation and position 2-Inter-implant distance 3-Geometry bevel position
[25] Pan et al., 2022	In-vitro	6	-	Zfx^TM^Intrascanmatchholder H4 and Zfx™ Evolution matchholder, Zimmer Biomet	Zfx Evolution plus+, Zimmer Biomet	Manufacturer’s instructions	-	CAD software	Geometry of SBs	Virtual alignment of SBs
[27] Kernen et al., 2022	In-vitro	6	Lateral incisor First premolar First molar	Camlog ø3.8 mm, CAMLOG Biotechnologies GmbH	E3, 3Shape	Manufacturer’s instructions	-	Autodesk Fusion 360	Different types of scan aids	irregular design in beige color
[26] Lee et al., 2021	In-vitro	6	Right second premolar First molar Second molar	1-PEEK 2-titanium (Myfit)	CS3600	Identica T500	-	DentalCAD	1-Implant angulation 2-SBs material	1-Mesially tilted distal implant 2-Titanium
[22] Tan et al., 2022	In-vitro	10	-	1-Nobel Procera Pos Locator 2-Sirona InPost 3-Amann Girrbach 4-Straumann CARES Mono 5-Core 3D 6-Straumann RC	1-Medentika L-Series 2-Straumann CARES Mono 3-Core 3D 4-Straumann RC	Manufacturer’s instructions	-	CMM	3D-positions of ISO and SBs	Positions of SBs and IOS
[17] Gherone et al. 2015	Clinical (14 patients)	4	Two axial Two titled	-	Lava COS	Manufacturer’s instructions	-	CAD software	1-Implant success 2-Implant survival 3-Restoration success	100% implant survival rate for all positioned implants
[16] Nagata et al., 2021	Clinical (30 patients)	5	Single molar Two distal Two mesial	Mono Scanbody RC, RN, Straumann®, Basel, Switzerland	Trios 3	Manufacturer’s instructions	-	CAD-CAM software	Digital and silicone impressions	Digital impressions using SBs
[29] Papaspyridakos et al., 2022	Clinical (35 patients)	4–6	Maxillary jaw Mandibular jaw	1-CARES Mono Scan body 2-ELOS multiunit scan body 3-ELOS Medtech; GM Mini Conical Abutment Scan Body 4-Cylindrical SRA scan bodies	TRIOS 3	Manufacturer’s instructions	-	CAD software	1-Scan body shape 2-Implant number	1-Scan body design 2- Number of implants

IOS: intra oral scanner; SBs: scan bodies; CHA-SB: combined healing abutment-scan body; CAD: computer-aided software; CAM: Computer-aided manufacturing; NS: not significant; MRM-DS: master reference model digital scan; CMM: coordinate measuring machine

### Quality assessment of studies

For each study, two independent reviewers (M.R., P.G.) assessed the risk of bias across the following domains: randomization process, deviations from intended interventions, missing study outcome data, measurement of outcomes, selection of the reported result, and overall risk of bias. Disagreements were resolved through discussion and consensus. The risk of bias was rated as low, high, or unclear for each domain, and an overall risk of bias was assigned for each study. Studies with a high or unclear risk of bias were excluded from the final analysis. To assess the impact of the high-risk studies on the overall results, an additional sensitivity analysis was carried out. The risk of bias quality assessment was conducted to ensure the validity and reliability of the included studies and to provide a clear understanding of the strength of the evidence base (Table 2).

**Table 2:** Example of ‘Risk of bias’ table for a single study

Entry	Judgement	Support for judgement
Sequence bias	Low Risk	Quote: “The method is detailed and repeatable.”
Blinding /performance bias	Low Risk	Quote: “The software is responsible for performing the comparison of scanning accuracy.
Blinding of outcome assessment	Low Risk	Quote: The operator and the data analyst are not the same person.
Incomplete outcome data addressed	Low Risk	0 missing from study groups
Selective reporting (reporting bias)	Low Risk	The rating scale for cognition listed in “Methods” is reported.

### Parameters of scan bodies influencing the accuracy outcome of intraoral scans

The ISB position was found to be a relevant factor affecting the accuracy of digital scans. An in-vitro study showed that distance (*P* < 0.001) and angular (*P* < 0.001) deviation values are parameters that significantly influence the trueness of ISB positions [18]. In addition, it has been reported that accuracy is unaffected by whether the palatal area of a maxillary scan was stitched or unstitched [18].

Additionally, in-vitro studies revealed that the orientation of the bevel on ISBs (the angle at which the scan body’s bevel is positioned), their placement within the dental arch, and implant angulation significantly influenced the precision of digital scans. Notably, a considerably higher level of accuracy was achieved when the implant was positioned lingually, as opposed to random, distal, mesial, or facial locations. The results demonstrated that the lingually positioned bevel exhibited distinct differences in linear measurements compared to other orientations (F = 7.92, *P* < 0.001), with an explanation of 2.80% of the variation [19].

A study on a dentate maxillary model using a combined healing abutment-scan body (CHA-SB) system and implants at three different sites reported that implant location could affect scan accuracy (trueness: *P* < 0.001, precision: *P* < 0.020). This study also evaluated whether a different operator could affect scan precision. However, the effect of the operator on scanning accuracy was found to be insignificant (*P* > 0.051) [8]. Using a similar CHA-SB system, four types of scan patterns were investigated. The results of this in-vitro study showed that the scan accuracy could be affected by the scan pattern selected. It was evident that the scan pattern exerted a significant influence on precision, particularly evident when considering angular deviation data (F = 6.227, df = 3, *P* = 0.002) [20].

A study on a master cast indicated that the accuracy of digital impressions is not related to angulation and implant depth. Interestingly, inexperienced operators performed better in this study, and camera position was one of the key factors that could improve accuracy [9].

In another ex vivo investigation, the primary aim was to evaluate the accuracy of five intraoral scanners in replicating ISBs and soft tissues within an edentulous maxilla, considering the influence of operator experience. The outcomes exhibited notable disparities in implant platform deviation between inexperienced and experienced operators following complete surface alignment. It is noteworthy that after alignment of the ISBs, no significant inter-operator variation was observed for the selected scanners. The scanner rankings displayed variability based on operator experience. Furthermore, the study uncovered a tendency for mucosal alignment to overestimate the platform deviation. These findings emphasize the critical role of operator expertise and meticulous scanner selection in achieving precise and reliable intraoral scanning outcomes for edentulous cases [21].

Trials evaluating the 3D positional accuracy of ISBs and IOS devices reported that the selected system could significantly affect the 3D positional accuracy. Six types of ISBs—Straumann RC, Core 3D, Straumann CARES Mono, Amann Girrbach, Sirona InPost, Nobel Procera Pos Locator—and four kinds of IOS devices—Straumann RC, Core 3D, Straumann CARES Mono, Medentika L-Series—were utilized. Straumann RC demonstrated the lowest accuracy for both ISBs and IOS [22].

Five types of ISB systems—AF (IO-Flo; Dentsply Sirona), NT (NT-Trading GmbH & Co KG), DE (DESS-USA), C3D (Core3Dcentres), and ZI (Zimmer Biomet Dental)—and four types of scanning techniques—no modification, glass beads, pressure indicating paste, and floss—were evaluated in an in-vitro model. As a result, the authors demonstrated that both ISBs and scanning techniques could significantly affect the accuracy of digital implant scans [23].

The accuracy of using four different types of IOS devices in an in-vitro model was investigated. Primescan and iTero devices showed superior digital scans with slight errors than Medit i500 and Vatech EZ scans (*p* < 0.05) [24].

Another in-vitro study used dome-shaped and cuboidal ISBs on a master model of an edentulous maxilla. The authors stated that the virtual alignment of ISBs could significantly affect the precision of digital scans (up to ∼ 30 μm/0.09°). The cuboidal ISBs in this study demonstrated larger deviations rather than dome-shaped ones [25].

ISB material and implant angulation were investigated in an in-vitro model. The results showed that titanium ISBs outperformed polyetheretherketone ISBs in terms of accuracy. In terms of angulations, mesially tilted distal implants exhibited better accuracy regardless of the type of intra-oral scanners [26].

Another in-vitro study using an ISB on a single implant in the right first molar position showed that the chosen measurement technique could affect the accuracy of digital scans. Three experienced operators performed evaluations using two different approaches: circle-based and point-based. Results displayed that the circle-based method had a significantly higher deviation than the point-based technique (*P* = 0.001) [10].

Scan aids can help to improve the accuracy of implant scans. Various designs - irregular, square, circular - and materials - white, gray, beige - of scan aids have been studied in-vitro. Findings showed beige color and irregular design have the highest precision, but their poor strength hinders the clinical use of this type. The clinically applicable form was gray in color and irregular in design [27].

One in-vitro study compared the accuracy of digital and conventional implant impressions. No significant differences in accuracy were found when scanning short spans, but when scanning long spans, digital impressions were significantly less accurate compared to traditional analog impressions. These results suggest that the scan span and implant position should be considered when choosing between digital and conventional impressions [28].

Clinical studies on the impact of ISBs on the scan outcome are scarce. The following three clinical trials were conducted with differing objectives related to ISBs. A recent clinical trial of 30 patients evaluated the accuracy of digital versus conventional impressions. The study demonstrated that digital scanning and the use of ISBs could potentially facilitate implant restorative treatment for practitioners and patients. Yet ISB misfit can occur based on the location of the respective implant. The lowest misfit was found for single molar implants (40.5 ± 18.9 μm) and the highest for distal three-unit implants (80.3 ± 12.4 μm) [16]. In a clinical study of 14 patients (8 women and 6 men), Gherlone et al. evaluated the survival rate of implants with digital impressions. After a follow-up period of 6–12 months, the survival rate was 100% for all implants examined. This study suggests that digital impressions provide accurate models that facilitate prosthetic work and satisfy the dental team [17].

In a retrospective clinical investigation of 35 patients, the effect of ISB design and number of implants were evaluated. First, the study showed that digital scans resulted in an acceptable fit of the implant superstructure with an accuracy of 86.70%. Second, the influence of the ISB design (*P* = 0.005) and the number of implants (*P* = 0.039) on the accuracy of fit was significant. Cylindrical ISBs on 4 implants exhibited better accuracy than polygonal-shaped ISBs [29].

## Discussion

It should be emphasized that the scope of this study is limited to the analysis of the performance of ISBs, independent of other variables associated with intra-oral scanning. While comparative data between digital impressions using ISBs and conventional impression techniques is still limited, available data suggest that ISBs, depending on their design and material, have a satisfactory level of accuracy, as well as favorable patient preference and time efficiency [30, 31]. Clinical findings highlight the precision and performance of digital impressions capturing with ISBs, demonstrating their beneficial impact on the workflow in implant rehabilitation [17]. Among the factors evaluated, cylindrical ISBs demonstrated a higher accuracy compared to polygonal ones. Superstructures supported by four implants exhibited better fit than those supported by six fixtures. However, it should be noted that this particular study had a limited sample size, which may constrain the generalizability of the results. The assessment of fit was based on the subjective evaluation by two prosthodontists, introducing potential bias. Other variables that may affect ISB accuracy, such as implant angulation, inter-implant distance, and model printer characteristics, were not evaluated.

The results of in-vitro studies indicate that implant position and ISB position can significantly affect the accuracy of digital scans. Regarding the position of the bevel geometry, the bevel’s orientation on the ISB directly influences scan accuracy, especially demonstrating higher precision when the implant is positioned lingually [19]. The statement on the angulation of implants contributing to more accurate scan results is clarified. It’s not solely about angulation but also about reducing the mesiodistal distance between implants in the edentulous region [26]. Controversy remains as to whether operator skill can influence scan accuracy. In scanners with lower inherent variability, operator experience significantly influenced accuracy, favoring experienced operators. Notably, the iTero system revealed variability among individuals rather than experience levels. Surprisingly, inexperienced operators achieved superior mean values and variation compared to experienced counterparts [9, 20]. Furthermore, while operator experience showed an improvement in the accuracy of the edentulous mucosa, it did not significantly affect implant platform linear deviation [21].

Moreover, the study concluded that mucosal alignment tended to overestimate platform deviation, and the trueness of complete-arch implant scanning varied among tested intraoral scanners. The type of ISB and intraoral scanner selected, as well as the scanning technique and patterns have an impact on the accuracy of the resulting digital scans. Every intraoral scanner (IOS) possesses the capability to create a digital impression of complete implants in an in vitro setting, aligning with the average misfit value. Nevertheless, upon conducting a 3D distance analysis, it was observed that only the Primescan and iTero exhibited minimal systematic error sources [22–24]. Notably, the ZI scan body exhibited considerably lower distance deviation, whereas using splinting scan bodies with floss resulted in a marked increase in distance deviation [20, 23]. Regarding ISB characteristics, the shape and the material have a noticeably influence on the digital transfer accuracy. Dome-shaped ISBs compared to cuboid-shaped as well as titanium ISBs compared to polyetheretherketone (PEEK) resulted in higher accuracy of the respective first-mentioned variants [25, 26]. High precision digital scanning has been shown to be directly dependent on the geometry and surface texture of the selected ISB. Sharp edges can cause significant noise that ultimately reduces the accuracy of the final digital scan [25]. Measurement techniques and scanning aids are other factors that may influence the precision of ISBs. The utilization of a point-based technique might be favored in research investigations focusing on the scan accuracy of implants due to its superior reliability compared to the circle-based technique [10, 27, 32]. As a limitation of these in vitro results, it should be noted that in a clinical setting, saliva, moisture, and oral conditions might further affect the accuracy of ISBs [25, 26].

Future clinical research with larger cohorts and objective evaluation methods are needed to validate these findings and fully investigate the impact of various factors on the transfer accuracy of ISBs during digital impression taking. The discussion of factors influencing the performance of ISBs in digital scans can be structured as followed to enhance clarity:

- **Scan Body Design**:

The geometric features of scan bodies, including their shape, bevel geometry, and surface texture, have a profound impact on scan accuracy. Research suggests that the bevel’s orientation, particularly when the implant is positioned lingually, contributes to higher precision. Additionally, dome-shaped ISBs have demonstrated superior accuracy compared to cuboid-shaped counterparts, emphasizing the significance of their geometric design.

- **Scan Body Material**:

Beyond geometric considerations, the material composition of scan bodies plays a crucial role in scan accuracy. Variations in materials, such as titanium versus polyetheretherketone (PEEK), have been identified as influencing factors.

- **Body Fit**:

The fit of scan bodies, influenced by factors like mucosal alignment, can impact platform deviation and, consequently, accuracy. While operator experience shows an enhanced accuracy in the edentulous mucosa, it does not significantly affect implant platform deviation. Further studies may provide insights into optimizing the interplay between fit and operator proficiency.

- **Implant Position and Angulation**:

Precise implant positioning and angulation are critical considerations for scan accuracy. Notably, the angulation of implants, can contribute to change the scan results.

- **Operator Skill**:

The influence of operator skill on scan accuracy remains a subject of controversy. Conflicting viewpoints from studies underline the need for a nuanced understanding of the specific aspects of operator proficiency that may impact scan outcomes.

- **Type of ISB, Intraoral Scanner, and Scanning Strategy**:

The choice of scan body type, intraoral scanner, and scanning technique significantly affects scan accuracy. Optimal combinations of these elements remain a subject for exploration, urging further research to identify strategies that enhance the precision of digital scans.

- **Measurement Techniques and Scanning Aids**:

Various measurement techniques and scanning aids contribute to the precision of intraoral scans. Furthermore, it’s crucial to acknowledge that clinical conditions, including saliva, moisture, and oral factors, may introduce additional complexities not fully captured in in vitro settings.

### Limitations and potential sources of bias

While this systematic review provides insights into the factors influencing the performance of implant scan bodies (ISBs) in digital scans, it is essential to acknowledge certain limitations that may affect the interpretation of the results.

- Study Diversity: The included studies exhibit variations in methodologies, sample sizes, and outcome measures, contributing to heterogeneity across the literature. This diversity may introduce challenges in directly comparing study findings.
- Limited Clinical Evidence: A predominant portion of the identified studies is in vitro investigations, which may not fully capture the complexities introduced by clinical conditions such as saliva, moisture, and oral factors. The translation of in vitro findings to real-world clinical scenarios requires cautious consideration.
- Scope of Analysis: This review focuses on the analysis of ISB performance. While this scope aligns with the specific objectives, it is crucial to recognize that the broader context of digital impression techniques encompasses multifaceted considerations.

Acknowledging these limitations is imperative for a nuanced interpretation of the findings, and future clinical research with larger cohorts and objective evaluation methods are needed to validate these findings and fully investigate the impact of various factors on the transfer accuracy of ISBs during digital impression taking. To advance the field, it is imperative to emphasize the adoption of standardized methodologies in upcoming studies. The implementation of standardized approaches will not only ensure robust research outcomes but also facilitate comparability across different research endeavors, promoting a more comprehensive understanding of intraoral scan body performance.

## Conclusion

Within the limits of the present systematic review the following conclusions can be drawn.

1. While intraoral scanning using implant scan bodies (ISBs) provides commendable accuracy in capturing implant positions, this conclusion may hold true primarily for single and short-span scenarios. The efficacy of this method in extensive complete-arch situations requires further exploration to account for potential challenges and variations.
2. A number of features such as the ISB position in the dental arch, its design, shape, material, color, and the manufacturing system of ISBs can influence the accuracy of the virtually generated scan.
3. The type of implant scan bodies (ISBs) and the choice of intraoral scanner (IOS) and scanning strategy play pivotal roles in determining the accuracy of resulting digital scans. A nuanced understanding of which ISB types and scanning combinations yield superior results is essential for practitioners seeking optimal outcomes.
4. The role of operator skill remains a point of discussion, requiring a more in-depth exploration of the factors contributing to scan accuracy.
5. Clinical data evaluating the accuracy of ISBs in patients are limited.

In conclusion, while this systematic review sheds light on critical factors influencing intraoral scanning accuracy in implant dentistry, it is imperative to acknowledge the scope and limitations of the current evidence. The dynamic nature of intraoral conditions, combined with the evolving landscape of scanning technologies, emphasizes the need for ongoing research and clinical validation.

### Electronic supplementary material

Below is the link to the electronic supplementary material.

Supplementary Material 1

## Data Availability

The data sets used and analyzed during the current study are available from the corresponding author on reasonable request.

## Declarations

### ISB systems and IOS devices in terms of brand name, manufacture, location, and country

Straumann CARES Mono Scan body for implant-level scanning (REF 025.4915) Institut Straumann AG, Basel, Switzerland.

Core 3D Scan body, Straumann Bone level RC (compatible) (REF 2077), Institut Straumann AG, Basel, Switzerland.

Straumann RC Scan body (REF 025.4905), Institut Straumann AG, Basel, Switzerland.

Amann Girrbach Scan body for Range 3 Kit B (REF 792322), Amann Girrbach, Mäder, Austria.

Sirona InPost for 2-CONnect KS61 (REF 6551639, REF 2-CONnect L810M), Dentsply Sirona Implants, Mölndal, Sweden.

Nobel Procera abutment Pos Locator for Straumann RC BL (REF 35564), NobelBiocare, Zurich, Switzerland.

Medentika L-Series Scan body Second Generation (REF L1420), Institut Straumann AG, Basel, Switzerland.

AF (IO-Flo; Dentsply Sirona), Dentsply Sirona Implants, Mölndal, Sweden.

NT (NT-Trading GmbH & Co KG), NT-Trading Verwaltungs-GmbH, Karlsruhe, Germany.

DE (DESS-USA), DESS Dental Smart Solutions, Reno, Nevada, USA.

C3D (Core3Dcentres), Core3dcentres®, Melle, Germany.

ZI (Zimmer Biomet Dental), ZimVie, Florida, USA.

## Abbreviations

3D: Three Dimensional
CAD/CAM: Computer-Aided Design/Computer Aided Manufacturing
CHA-SB: Combined Healing Abutment-Scan Body
IOS: Intraoral Scanner Completion
ISB: Implant Scan Body
PICOT: Population, Intervention, Comparison, Outcome, Time frame
PRISMA: Preferred Reporting Items for Systematic Reviews and Meta-analyses
RCT: Randomized Clinical Trials
ROBINS-I: Risk Of Bias In Non-randomized Studies - Of Interventions
